# Ultrasound power and irrigation volume in different lens opacity grades: comparison of femtosecond laser-assisted cataract surgery and conventional phacoemulsification

**DOI:** 10.6061/clinics/2019/e1294

**Published:** 2019-10-23

**Authors:** Guilherme A. Horta, Rogério C. Horta, Kátia Steinfeld, Camila R. Koch, Glauco R. Mello, Newton Kara-Junior

**Affiliations:** I Centro de Estudos e Pesquisas Oculistas Associados, Rio de Janeiro, RJ, BR; II Instituto de Olhos Dr. Rogerio Horta, Rio de Janeiro, RJ, BR; III Université de Lausanne, Lausanne, France; IV Departamento de Oftalmologia, Faculdade de Medicina FMUSP, Universidade de Sao Paulo, Sao Paulo, SP, BR; V Universidade Federal do Parana (UFPR), Curitiba, PR, BR

**Keywords:** Femtolaser Cataract Surgery, Ultrasound Energy, Irrigation Volume, Balanced Salt Solution Use

## Abstract

**OBJECTIVES:**

To compare the amount of ultrasound energy and irrigation volume in conventional phacoemulsification cataract surgery *versus* femtosecond laser-assisted phacoemulsification at different nuclear-cortical cataract grades.

**METHOD:**

This was a prospective, consecutive, investigator-masked nonrandomized parallel cohort study. Patients were divided into 4 groups (Phaco1, Phaco2, Femto1 and Femto2) according to the surgical technique (conventional phacoemulsification [Group Phaco] or femtosecond laser-assisted cataract surgery [Group Femto]) and the Lens Opacity Classification System III (LOCS) grade (LOCS<11 [group 1] or LOCS≥11 [group 2]). The measured outcomes were effective phacoemulsification time (EPT), indicating the ultrasound energy, and balanced salt solution (BSS) use, indicating the irrigation volume, to indirectly estimate the damage to the corneal endothelium caused by the cataract surgery.

**RESULTS:**

A total of 160 eyes from 109 patients were included: 87 eyes in Group Phaco, 73 eyes in Group Femto, 76 eyes in group 1 and 84 eyes in group 2. The EPT mean in Femto1 was 53% less (2.73±1.88, 0.1 to 8.65) than that in Phaco1 (5.80±2.86) (*p*=0.00) and in Femto2 (8.38±9.32) was 33% less than that in Phaco2 (12.55±8.38) (*p*=0.00). No significant differences in mean LOCS grades between the Phaco1 (8.21±1.44) and Femto1 (7.90±1.90) groups (*p*=0.73) or between the Phaco2 (13.15±2.55) and Femto2 (12.72±2.18) groups (*p*=0.95) were found. There were no significant differences in the mean BSS use between the Phaco1 (55.73±12.45) and Femto1 (59.37±10.93) groups (*p*=0.48) or between the Phaco2 (64.34±21.00) and Femto2 (65.71±17.60) groups (*p*=0.47).

**CONCLUSIONS:**

Compared to conventional phacoemulsification at different nuclear-cortical cataract grades, femtosecond laser-assisted cataract surgery provides an EPT reduction but does not influence the BSS use.

## INTRODUCTION

Cataracts remain the most common potentially reversible cause of blindness, with an estimated 19 million phacoemulsification surgeries performed per year and a prospective 32 million surgeries projected in 2020 (1-3). Due to the increase in the population’s life expectancy, it is essential to preserve corneal endothelial cells for long-term visual outcomes after cataract surgery (4-6). The proper functioning of these cells maintains the transparency of the cornea and avoids stromal edema (7-10).

Studies have shown that reducing the use of ultrasonic energy during phacoemulsification cataract surgery and reducing the volume of balanced salt solution (BSS) used can decrease endothelial cell loss (11-18). There is a shorter effective phacoemulsification time (EPT) and less endothelial cell loss in surgery assisted by a femtosecond laser than in conventional manual phacoemulsification (10-19). Since the femtosecond laser is still a new technology, long-term studies are necessary to analyze its real impact because innovations are constantly emerging, and many variables can influence endothelial cell loss (7-10). The present study compared the ultrasound power and BSS use in conventional manual phacoemulsification surgeries with those in femtosecond laser-assisted surgery.

## MATERIAL AND METHODS

### Study population

This investigation was a prospective, consecutive, investigator-masked, nonrandomized parallel cohort study performed at a single center. The study was performed in accordance with the Declaration of Helsinki. An appropriate written informed consent form was signed by all patients prior to surgery. Consecutive patients who had a cataract surgery indication and were older than 50 years were enrolled in the study. Eligible patients were included in the study after undergoing an extensive preoperative assessment. One hundred and sixty (160) patients were recruited between October 2015 and April 2016, and the sample calculation was based on similar studies comparing conventional phaco and femto cataract surgeries. All operations were performed by one experienced surgeon (IORH). Patients were excluded from the study if they had any preoperative ocular comorbidity (e.g., glaucoma, cornea guttata, corneal opacity), previous eye surgery, a posttraumatic cataract or intraoperative complications.

Each cataract was graded with the Lens Opacity Classification System (LOCS) scale by a masked investigator using a slit lamp. According to the LOCS, nuclear opalescence (NO), nuclear color (NC) and cortex (C) grading were considered in the sum (NC+NO+C≥11 or <11), and patients were divided into groups according to their LOCS grade: LOCS<11 (group 1) or LOCS≥11 (group 2). All eligible patients underwent a preoperative evaluation to determine the possibility of undergoing pretreatment with a femtosecond laser, with the added value being funded by the patient. Subsequently, they were divided according to the proposed surgical technique, specifically, conventional phacoemulsification (Group Phaco) or femtosecond laser-assisted cataract surgery (Group Femto).

### Data collection

The data collected included sex, laterality, patient age at the time of cataract surgery, LOCS grade, the type of intraocular lens (IOL) implanted, the surgical technique, the EPT and the total BSS used in the surgery. The EPT represents the cumulative ultrasound energy used intraoperatively during the phaco procedure. The elapsed phaco is used to calculate the total energy delivered into the eye in one surgery. The BSS volume is an important fluid parameter for balancing aspiration and maintaining the anterior chamber during surgery. It measures the irrigation volume used during surgery and can be used to calculate the volume escaping through the surgical wounds. These parameters were informed by the Infiniti^®^ Vision System (Alcon Laboratories, Inc.) in each surgery and were used to indirectly estimate the cataract surgery damage to the corneal endothelium. At the end of each surgery, these data were checked and then compared according to the technique of the cataract surgery performed and the LOCS grades.

### Surgical technique

All operated eyes received topical moxifloxacin 30 minutes before the surgery. The pupils were dilated with phenylephrine, tropicamide and cyclopentolate. Anesthesia was used for conscious sedation and with topical 0.5% proparacaine hydrochloride and 0.75% bupivacaine hydrochloride, except in sensitive patients or those with very mature cataracts, who received peribulbar anesthesia with 2% lidocaine in combination with Hyalozima 50 IU/ml.

In Group Phaco, manual temporal incisions were made in the clear cornea; the main incision was 2.3 mm and the secondary incision was 1.2 mm, both in three planes and self-sealing. In group B, the LensX^®^ (Alcon Laboratories, Inc.) interface contact lens (SoftFit^®^) system was used for coupling to the vacuum and ocular fixation. Approximately 5 minutes before the use of the laser, a drop of naphazoline hydrochloride 0.025% to 0.3% and pheniramine maleate (Claril, Alcon Laboratories, Inc.) was administered to decrease subconjunctival bleeding due to eye suction. The surgeon’s predefined preferences for the use of the femtolaser were as follows: anterior capsulotomy of 4.7 mm; temporal clear corneal incisions (main - 2.3 mm; secondary - 1.2 mm); lens cube fragmentation pattern with 14 μJ, anterior capsule distance of 500 μm and posterior capsule distance of 800 μm, 20 μm spacing between the laser application points, 40 μm spacing between the horizontal layers, and a treated area 6 mm in diameter. After treatment with the laser, the surgery was immediately followed by phacoemulsification and IOL implantation. In all patients, after removing the anterior lens capsule, a full hydrodissection was performed with an ophthalmic viscosurgical device (OVD). Sodium hyaluronate 1.0% (DisCoVisc, Alcon Laboratories, Inc.) was injected to fill the anterior chamber.

The same parameters of phacoemulsification (Infiniti^®^ Vision System, Alcon Laboratories, Inc.) were used for all groups: torsional ultrasound technology (Ozil^®^ pen, mini Kelman^®^ 30° flared tip) and linear mode. During phacoemulsification, the sculpture stage in the linear pulse mode had the following upper limits: a BSS bottle height equivalent to 100 cmH2O, and aspiration rate of 30 ml/min, a vacuum level of 100 mmHg, a torsional amplitude of 90%, and 45 pulses per second (pps). The chop phase, in linear Burst mode, had the following upper limits: a BSS bottle height equivalent to 110 cmH2O, an aspiration rate of 30 ml/min, a vacuum level of 300 mm Hg, and a torsional amplitude of 90% (30 ms on and 10 ms off). The technique used was called &quot;Stop and Chop&quot;, which started with the sculpture of a central channel in the nucleus of the lens and then ended with the division with the phaco pen and a hook on the secondary incision. Emulsification was performed a half at a time, with the most suitable technique for the case. Cortical removal was conducted after the complete removal of the nucleus, and then the OVD was again injected into the anterior chamber. A foldable acrylic aspheric monofocal or diffractive multifocal IOL (AcrySof IQ and ReSTOR +3.0 [Alcon Laboratories, Inc], Tecnis monofocal and Tecnis multifocal +3.25 [Johnson & Johnson, NJ, USA], namely, a CT Asphina or an AT Lisa [Carl Zeiss Meditec AG]), was implanted into the capsular bag through the main incision, and then the OVD was removed. Corneal incision sealing was performed by stromal hydration, and if there was leakage, a bandage contact lens was used for one week.

### Statistical analysis

Statistical analysis was performed with SPSS version 21.0 (SPSS, Inc., Chicago, IL) software. In comparative tests between the groups, a 95% significance level was used. The Mann-Whitney test was used to compare the LOCS and EPT grades between Groups Phaco and Femto. The BSS values were evaluated by *t* tests.

## RESULTS

**Figure 1:**
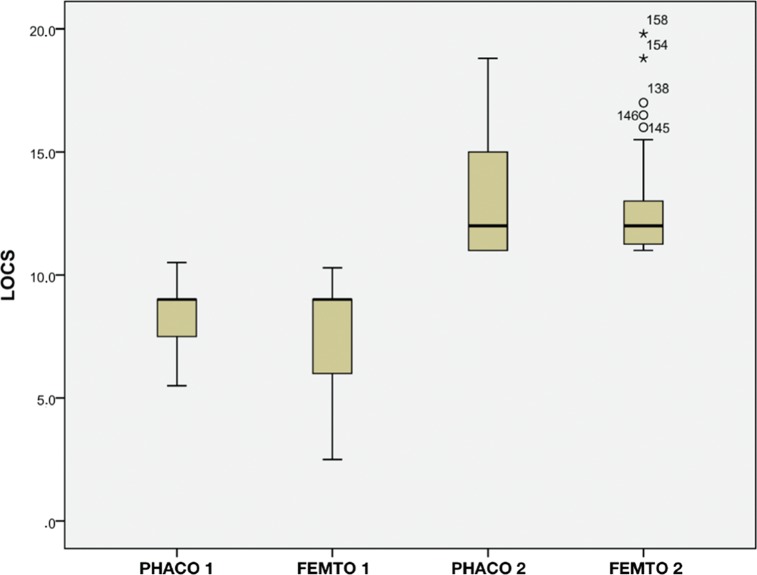
LOCS by Group. LOCS=Lens Opacity Classification System III.

This prospective study included 160 eyes of 109 patients. There were 57 women (52.2%). Group Phaco (87 eyes) underwent conventional phacoemulsification, and Group Femto (73 eyes) underwent femtolaser-assisted cataract surgery. Eyes with cataracts graded at LOCS<11 were assigned to group 1 (76 eyes). Those with cataracts graded at LOCS≥11 were assigned to group 2 (84 eyes). Table 1 shows the number of eyes, average LOCS grade, EPT and BSS use in each group and the *p*-value of the statistical comparisons between group 1 and group 2. There was no significant difference between the average LOCS grade of Group Phaco1 (8.21±1.44) and Group Femto1 (7.90±1.90, *p*=0.73) or between that of Group Phaco2 (13.15±2.55) and Group Femto2 (12.72±2.18, *p*=0.95). Figure 1 shows that the average LOCS grade in group 1 was significantly lower than that in group 2. Therefore, it was possible to compare the groups (Phaco1 *vs*. Femto1 and Phaco2 *vs*. Femto2) without a bias in the results related to cataract grade.

**Figure 2:**
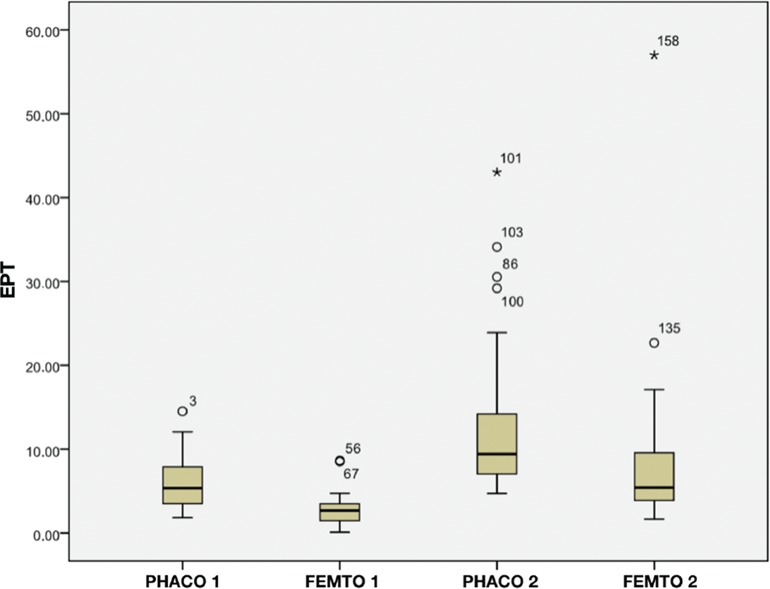
EPT by Group. EPT=Effective phacoemulsification time.

**Figure 3:**
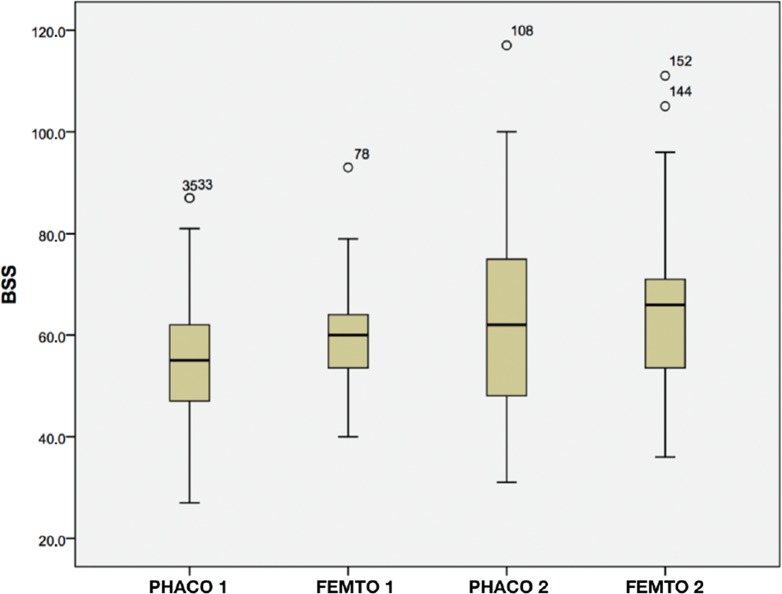
BSS by Group. BSS=Balanced salt solution.

There was a statistically significant reduction of 53% in the average EPT between Groups Phaco1 (5.80±2.86, 1.82 range 14.52) and Femto1 (2.73±1.88, 0.1 to 8.65) (*p*=0.00), and a 33% reduction between Groups Phaco2 (12.55±8.38, 4.73 to 43.03) and Femto2 (8.38±9.32, 1, from 66 to 57.00) (*p*=0.00). Figure 2 shows the EPT in each group: Phaco1 (5.33), Femto1 (2.65), Phaco2 (9.43), and Femto2 (5.44). There were similar EPT values in Groups Phaco1 and Femto2. Therefore, with similar degrees of LOCS classification, the Femto Groups had lower average EPTs than the Phaco Groups. There were no complications in either group. The results of BSS use show that the Phaco Groups had lower means than the Femto Groups when similar degrees of LOCS classifications were compared; however, there was no statistically significant difference in the BSS use between the groups (see Figure 3, Group Phaco1: 55.73±12.45 *vs*. Group Femto1: 59.37±10.93, *p*=0.48, and Group Phaco2: 64.34±21.00 *vs*. Group Femto2: 65.71±17.60, *p*=0.47).

## DISCUSSION

The femtosecond laser was introduced in ophthalmology in 2001 for the purpose of creating a lamellar flap during laser in situ keratomileusis (LASIK). Subsequently, its use has been expanded to cataract surgery to create corneal incisions, perform capsulotomy and produce nucleus fragmentation (8,9). In cataract surgery, endothelial cells can be damaged by the contact of instruments or the IOL, irrigation turbulence, movements of the nucleus fragments, mechanical trauma of sonic waves and injury due to heat (7-10). Moreover, investigators have reported that a high nucleus grade, high infusion volume and high ultrasound energy are some of the variables correlated with the percentage of endothelial cell loss (7,20,21). In our study, two surgical techniques were compared in corticonuclear cataracts with different densities, and the EPT and BSS use were compared between the groups.

The phacoemulsification time is the most significant factor for endothelial cell damage (4). Abell et al. (22) showed a significant reduction in endothelial cell loss and corneal edema in the early postoperative period (1 day and 3 weeks) with the femtosecond laser. However, they showed no statistical evidence that the decrease in ultrasonic energy by the femtosecond laser reduced the endothelial cell loss at 6 months of surgery, except in cases where the EPT reached zero or in cases of femtosecond laser-assisted procedures without corneal incisions, showing that a large reduction in the energy used can bring benefits to the corneal endothelium. A possible explanation for laser corneal incisions causing greater endothelial cell damage than manual incisions could be due to the location of the corneal incisions (closer to the corneal center) and architecture. Moreover, even though the reduction in endothelial cell loss at the beginning of the postoperative period was the only clinically significant result, a reduction in endothelial cell loss can result in a rapid visual recovery (22).

The presented results are in line with those of other studies. Many contributions to the femtosecond laser cataract surgery came from Conrad-Hengerer et al. A study in 2012 that graded cataracts with the LOCS demonstrated a mean of 3.4±0.9 in the femtosecond laser group and 3.1±0.9 in the conventional group, and the average EPT was 0.16 (±0.21) and 4.07 (±3.14), respectively (9). There was a significant reduction in the EPT in the femtosecond laser group. Additionally, in 2012, another study compared fragmentation patterns with laser femtosecond in 160 eyes and showed a significant reduction in the ultrasound time in the group with a distance of 350 μm between the lines through the application of the laser (2.05±3.08 seconds) than in the group with a distance of 500 μm (5.85±5.55 seconds) as well as a statistically significant reduction in the EPT (0:03±0:05 seconds 0:21±0:26 seconds, respectively) (23). In 2015, the EPTs in femtosecond laser-assisted surgery groups and conventional cataract surgery groups including brunescent cataracts with LOCS NO3 and NO5 (Phaco1 *vs*. Femto1 and Phaco2 *vs*. Femto2) were compared and showed no bias in the results related to cataract grade (24). The EPT in the femtosecond laser group was significantly lower than that in the conventional cataract surgery group for both grades, NO3 (0±0 seconds *versus* 1.38±1.35 seconds, respectively) and NO5 (1.35±1.64 seconds *versus* 6.85±3.83 seconds, respectively) (*p*<0.001). The average EPT in the femtosecond laser group with LOCS NO5 was lower than that in the conventional surgery group with LOCS NO3, with a significant difference between groups (*p*=0.013). Daya et al. (25) showed an EPT of 9.89±5.32 seconds in the conventional surgery group and an EPT of 8.58±4.66 seconds in the femtosecond laser group, with a reduction of 13.2% (*p*=0.044) (24).

Reddy et al. (26), with the VICTUS^®^ platform (Bausch + Lomb, New Jersey, USA), found a significantly lower EPT in the femtosecond laser group (5.2±5.7 seconds) than in the conventional surgery group (7.7±6.0) (*p*=0.025), and the BSS use was slightly higher in the femtosecond laser group (86.0±25.8 ml) than in the conventional surgery group (84.6±29.6 ml), but without a significant difference. The BSS use in our study was similar to that of Reddy et al., with no statistically significant differences between the groups, although the femtosecond laser group presented more BSS use than the conventional surgery group. To explain these results, we believe that the femtosecond laser-assisted group experienced less manipulation with the phaco tip and more manipulation with the aspiration pen, possibly because of an adventitious cortex due to laser bubbles, which caused great difficulty in hydrodissection. Therefore, fracture with a laser is more effective than the conventional technique since it consumes less EPT and the BSS is equivalent; moreover, due to the increased manipulation in the cortex aspiration phase but not in the phacoemulsification phase, regardless of BSS use, these results may be clinically significant. Nevertheless, additional randomized studies are required to establish the real, clinically significant benefits of femtosecond laser-assisted cataract surgery compared to conventional techniques. This study had limitations such as nonrandomization, a lack of safety data, a lack of postoperative results, and issues with refraction and reproducibility of the femtolaser technology; however, other studies cover these topics.

## CONCLUSION

Cataract surgery assisted by a femtosecond laser is comparable to the conventional technique and can reduce postoperative complications. Compared to conventional cataract surgery, femtosecond laser-assisted surgery for different levels of corticonuclear cataracts significantly reduces the EPT and does not change the BSS use. Therefore, further research is needed to quantify the EPT reduction necessary to effectively protect the corneal endothelium and decrease cell death compared to these outcomes in the conventional technique. The technology and surgical techniques must continue to evolve to optimize cataract surgery results and lead to better incisions as well as reduced BSS use and EPTs.

## AUTHOR CONTRIBUTIONS

Horta GA provided substantial contributions to the conception and design of the study, acquisition, analysis and interpretation of data, drafting and critical revision of the manuscript for important intellectual content and approval of final version of the manuscript. Kara-Junior N provided substantial contributions to the conception and design of the study, drafting of the manuscript and approval of the final version of the manuscript. Horta RC and Steinfeld K provided substantial contributions to the acquisition of data, critical revision of the manuscript for important intellectual content and approval of the final version of the manuscript. Koch CR and Mello GR provided substantial contributions to the conception and design of the study, analysis and interpretation of data, drafting and critical revision of the manuscript for important intellectual content and approval of the final version of the manuscript. All authors agreed to be accountable for all aspects of the work in ensuring that questions related to the accuracy or integrity of any part of the work are appropriately investigated and resolved.

## Figures and Tables

**Table 1:** Number of eyes and average LOCS grade, EPT and BSS used in each group as well as the *p*-value of the statistical comparisons between groups 1 and 2.

Groups	Phaco1	Femto1	*p-*value	Phaco2	Femto2	*p-*value
Eyes (n)	42	34	-	45	39	-
LOCS	13.15±2.55	12.72±2.18	0.95	8.21±1.44	7.90±1.90	0.73
EPT	5.80±2.86	2.73±1.88	0.00	12.55±8.38	8.38±9.32	0.00
BSS	56.09±12.12	59.82±10.75	0.48	63.53±19.44	65.71±17.60	0.47
